# Development of a Treatment Protocol for Cobra (*Naja naja*) Bite Envenoming in Dogs

**DOI:** 10.3390/toxins12110694

**Published:** 2020-11-02

**Authors:** Ranjith Adhikari, Lalith Suriyagoda, Amal Premarathna, Niranjala De Silva, Ashoka Dangolla, Chandima Mallawa, Indira Silva, Indika Gawarammana

**Affiliations:** 1 Department of Veterinary Clinical Sciences, Faculty of Veterinary Medicine and Animal Science, University of Peradeniya, Peradeniya 20400, Sri Lanka; adikari04@gmail.com (R.A.); niranjalad@yahoo.com (N.D.S.); adangolla@gmail.com (A.D.); mrckmallawa@yahoo.com (C.M.); indiradush@gmail.com (I.S.); 2 Department of Crop Science, Faculty of Agriculture, University of Peradeniya, Peradeniya 20400, Sri Lanka; lalith.suriyagoda@gmail.com; 3 Department of Veterinary Pathobiology, Faculty of Veterinary Medicine and Animal Science, University of Peradeniya, Peradeniya 20400, Sri Lanka; amaldharmapriya@gmail.com; 4 Department of Medicine, Faculty of Medicine, University of Peradeniya, Peradeniya 20400, Sri Lanka

**Keywords:** cobra bite, *Naja naja*, dogs, antivenom serum, clinical manifestations

## Abstract

There is limited information on clinical profiles, treatment, and management aspects of Indian cobra (*Naja naja*) bite envenoming in dogs in Sri Lanka. Dogs with cobra bites presented to the Veterinary Teaching Hospital (VTH), University of Peradeniya, were prospectively studied over a period of 72 months; local and systemic clinical manifestations and hematological abnormalities were recorded. We studied 116 cobra bite envenomings in dogs. A grading system was established using a combination of anatomical site of fang marks, as well as local and systemic clinical manifestations. Accordingly, treatment strategies were established using Indian polyvalent antivenom (AVS). Pain and swelling at the bite site were major clinical signs observed, while neurotoxic manifestations (mydriasis, wheezing, and crackles) were detected in most dogs. Leukocytosis was observed in 78% of them. Statistical analysis revealed that the grading scores obtained were compatible to initiate AVS administration according to the severity. The minimum number required was 2 AVS vials (range 2–12). Almost 20% of the dogs developed wheezing, crackles, hypersalivation, restlessness, and dyspnea as adverse reactions to AVS treatment. Necrotic wounds on bitten anatomical sites developed in 19% of the dogs and 2.5% developed acute kidney injuries as a consequence of envenoming crisis. Despite treatment, 3% of dogs died. No dry bites were recorded.

## 1. Introduction

**Figure 1:**
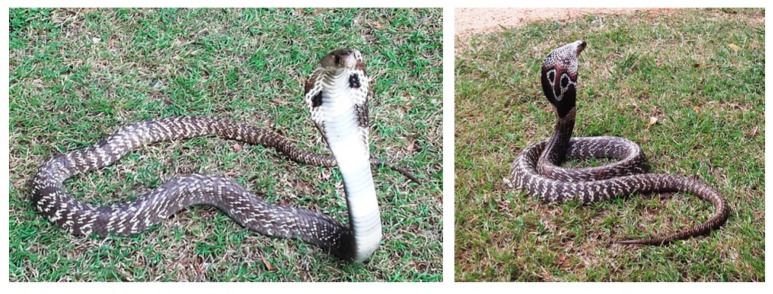
Adult specimens of *Naja naja* (Photo credit: Mr. Sanath Velarathne).

Snake envenoming is a leading reason for morbidity and mortality in the rural tropics around the globe [1]. Approximately 375 snake species around the world are venomous and are considered dangerous [2,3,4]. Although an accurate figure of the burden of global snakebites is unavailable, an estimate of 5.5 million annual snakebites across the globe is considered realistic [5]. Sri Lanka has one of the highest rates of snakebite incidence in the world. There are about 100 species of snake on the island [6,7]. However, the high morbidity and mortality from snake envenoming is due to snakebites in dogs in Sri Lanka, resulting from four species, namely, cobra (*Naja naja*), Russell’s viper (*Daboia russelii*), and hump-nosed viper (genus *Hypnale*) [8]. The spectacled cobra is the only member of the genus *Naja* reported in Sri Lanka (Figure 1) [9]. The body of this cobra is long and cylindrical and can be as long as 1.8 m. The color of the hood and body vary greatly according to the environment that they inhabit [9,10,11]. This snake is commonly seen in the ecosystems of coastal plains and in mountain regions at elevations up to 1500 m [12]. Records regarding envenoming in man are abundant: pain, swelling, blister formation, and hemorrhagic necrosis are the symptoms generally seen following a snakebite in man [13]. Severe localized tissue necrosis, pain, and swelling are produced due to the toxins contained in the cobra venom [14]. Bites by *N. naja* can cause significant local swelling and sometimes cause severe local necrosis in man and animals. Immediate severe local pain and blister formation, bleeding from the bitten site, as well as lymphadenitis have been observed [15]. Phospholipase A_2_ and cytotoxins are the principal toxic compounds in cobra venom, causing cardiotoxicity, myotoxicity, neurotoxicity, and hemolysis [16]. Anticoagulant factors present in cobra venom lead to destruction of tissue thromboplastin, which exerts an anticoagulant effect and causes bleeding [17]. Snakebite in animals generally occurs while grazing, hunting, or playing in gardens [18]. Despite the fact that snakebites in canine patients are not well documented, dogs are the commonest snakebite victims encountered in veterinary practice [18,19]. Clinical features of envenoming on admission depend on the dose and type of venom; the anatomical site and number of bites; the age, size, and health status of the victim; and the time between the bite and admission for veterinary care [20]. Irrespective of the wide variety of snakes, snake venoms share many common toxin groups, including neurotoxic PLA2 and three-finger toxins [21].

Antivenom (AVS) is the worldwide (universal) specific treatment option for patients with snakebite envenoming [22]. However, Sri Lanka currently depends on Indian polyvalent AVS, as it does not produce its own species-specific antivenom [23]. AVS-induced complications related to this have been reported frequently in human patients [24]. Though canine snake envenoming cases frequently present to veterinary practitioners in Sri Lanka, documented information on therapy and epidemiology of envenomation is sparse [25]. Therefore, this study was carried out with a view to document the local and systemic effect of *N. naja* envenoming in dogs and to draft a treatment and management plan for such individuals. Thus, we report the first detailed clinical study cases on cobra bite envenoming in dogs at the only available Veterinary Teaching Hospital (VTH) in Sri Lanka.

## 2. Results

**Figure 2:**
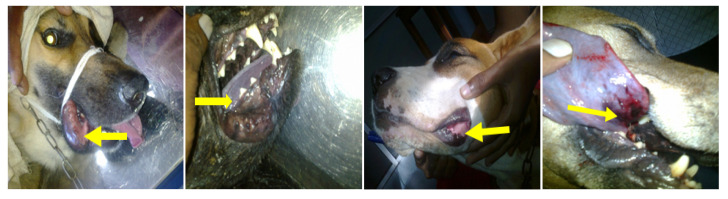
Fang marks of cobra bite envenoming of deceased dogs.

During the study period, 116 dogs were admitted due to cobra bite envenoming, of which 71 were males and 45 were females. Their median age was 4 years (interquartile range (IQR): 2–6.75 years). The median time to hospital admission was 2 h (IQR: 8.25 h) after the bite. The median hospital stay was 7 days (IQR: 10 days). The case fatality of the cobra-bite-envenomed dogs was 4.6% (*n* = 4). The median time to death was 16 h (IQR: 17.6 h). The site of the bite of all the dogs that died was oral cavity and all of them showed neurological manifestations, dilated pupils, cardiorespiratory manifestations, hypotension, wheezing, crackles, cyanosis, and dyspnea at the time of death. Figure 2 shows fang marks on dead dog patients. Two deaths (1.7%) out of four were reported due to AVS reaction, in which hypotension was observed. None of the dogs with fang marks inside the oral cavity survived.

The severity of the bites were graded as follows: a minimum 2–3 (8.6%) and a maximum of 15–17 (5.6%) (Table 1); accordingly, 8.6% of the dogs with the lowest severity were given 2 vials of AVS and 5.6% of the dogs that were severely envenomed were given 12 vials. The number of AVS vials given to the patients based on the visual symptoms (grading score) was appropriate, as the survival rate was similar in each group, ranging from mild to severe (*p* > 0.05). Therefore, irrespective of the severity of the symptoms, over 99% of the patients survived when this method was applied.

A total of 17% *(n =* 20) of the dogs developed wheezing, crackles, hypersalivation, restlessness, and dyspnea during and after administration of AVS. The minimum time taken to detect such adverse reactions was 15 min ($\bar{X}$ = 2 h). All of them were subjected to endotracheal intubation for assisted ventilation, continued for 2–6 h, in addition to treatment of anaphylactic reactions.

### 2.1. Clinical Manifestations of Cobra Bite Envenoming

**Figure 3:**
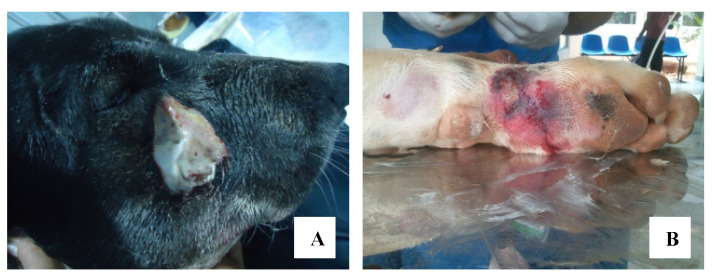
Development of necrotic wounds in different anatomical locations: (**A**) necrotic wound on right maxillary region; (**B**) necrotic wounds on medial side of the tarsometatarsal region.

The main clinical symptoms were noted locally around the site of the bite. Pain was seen in 97% of the dogs and mild swelling was seen in 51%, while 36% had severe swelling. Necrosis of the site was observed in 19% (Figure 3). Systemic manifestations were seen in 75%. These were mainly concerned with the nervous system (Table 2).

### 2.2. Laboratory Investigations of Hematological Parameters, Urinary System, Liver Function, and Coagulation Factors

The predominant laboratory abnormality at admission was leukocytosis, observed in 78% of patients (median: 34.61 × 10^3^/mL; IQR: 19.76 × 10^3^/mL). Leukocytosis was seen in 94% of patients at 24 h after admission. Thrombocytopenia was a notable abnormality at admission (median: 130.05 × 10^3^/mL; IQR: 64.8 × 10^3^/mL) and at 24 h after admission (Table 3 and Table 4).

Hematuria (dipstick reading and microscopic examination) and proteinuria were the renal abnormalities observed and their percentages on admission were 13% (*n* = 15) and 11% (*n* = 13), respectively. The number of patients with hematuria and proteinuria reduced to 6% (*n* = 7) at 24 h after admission (Table 3). All patients showed normal levels of total serum proteins and albumin, while none of them showed elevated transaminases (alanine aminotransferase (ALT) and aspartate aminotransferase (AST)). A prolonged partial thromboplastin time (PT) level of 11.5–30 s (median: 16.30; IQR: 10.35) was detected in 17% (*n* = 20) of patients and a prolonged activated partial thromboplastin time (aPTT) was detected in 25% (*n* = 29) of dogs (range 23.8–68 s). Clotting time was not affected in any of the patients. Within 24 h of administration AVS, PT and aPTT levels returned to normal levels in all the dogs

### 2.3. Observation of Development of Acute Kidney Injuries (AKI)

Continuous monitoring of patients revealed that there were 2.3% (*n* = 2) of patients with AKI. Both of these dogs exhibited proteinuria (++300 mg/dL), oliguria (0.33 mL/kg/h)/anuria, and urinary casts. Azotemia was detected by elevated blood urea nitrogen (BUN) (>147.85 mg/dL) and elevated serum creatinine (>3.59 mg/dL). Therefore, treatment for AKI was initiated with the appearance of clinical manifestations using the treatment schedules mentioned. The dogs recovered from the signs of AKI in 5 days after the treatments.

## 3. Discussion

In this study, a higher proportion of male dogs experienced cobra bite compared with female dogs. However, in humans, females are more prone to cobra bite envenoming since this snake naturally tends to live in close proximity to human settlements [1]. This study demonstrates the remarkable variation of cobra bite envenoming among dogs. All dogs admitted to VTH with cobra bite were envenomed; in contrast, dry bites are reported in about 20% of human cases [1]. Therefore, it can be inferred that cobra bites in dogs seem to be a result of provocation, rather than being incidental. Consequently, almost all of the dogs had local reactions such as inflammation, blistering, necrosis, and pain at the site of the bite. In vitro studies injecting *Crotalus atrox* metalloprotease, a venom protein, to the tibialis anterior muscle of mice showed that this toxin damages skeletal muscles by attacking the collagen scaffold and other important basement membrane proteins and prevents their regeneration through disruption the functions of satellite cells. Hence, excessive tissue/muscle damage can be seen in snakebite victims [26]. Reduction of extracellular matrix viscosity is caused by hyaluronidase and the digestion of collagen is caused by collagenase, leading to vascular leakage and severe local swelling. C3b (complement activating factor) binds to Bb factors and activates C5 convertase. This may form membrane attack complexes resulting in local inflammation and massive tissue damage at the bite site [27]. Further, necrotic wound development was observed at the anatomical sites where cobra fang marks were observed. Early surgical referrals are indicated for such patients in order to minimize the extent of necrotic trauma development due to snakebite envenoming [8]. Thus, snakebite envenoming is a medical emergency causing quick onset of both local tissue-destructive clinical manifestations and potentially lethal systemic hemorrhagic and neurotoxic pathologies [28]. Envenoming by *N. naja* causes a broad range of systemic toxicities, including neurotoxic, hemorrhagic, hemolytic, inflammatory, and necrotic effects on different organs, leading to multiple organ failure in humans [9]. Mydriasis, hypersalivation, recumbency, hyperesthesia, tachypnea, wheezing, crackles, and bradypnea were seen in the majority of envenomed dogs as neurotoxic clinical manifestations. Thus, snake-venom-induced paralysis that leads to progressive paralysis of the bulbar and respiratory muscles requires prompt airway assistance and mechanical ventilation to mitigate impending life-threatening consequences [5,29]. Close monitoring of patients’ vital signs of oxygen saturation and activities of the cardiovascular system using a pulse oximeter and other ancillary care is important to obviate impending critical situations. Protein kinase C, cardiotoxin, and cytotoxin, which are present in cobra venom, cause cardiovascular system abnormalities [30]. Hence, it is remarkable that cardiovascular toxicities such as cardiac arrhythmias, hypotension, and bradycardia were not detected in dogs with cobra bite envenoming in this study. Myotoxic elapid PLA_2_s and some viperid PLA_2_s in snake venom lead to acute kidney injury [31], and development of acute renal failure was observed in 2.3% (*n* = 2) of the dog patients. Therefore, dogs that are subjected to cobra bite envenoming should be monitored for their renal functions.

Leukocytosis is observed in humans with snakebite envenoming. This has also been observed in dogs [13,32]. However, the cause of leukocytosis following such snakebite envenoming is yet to be explored [13]. This parameter would have the potential to assess the severity of envenoming. Laboratory investigation of coagulation abnormalities has shown mild coagulopathy with elevated aPTT and PT in these patients. However, clotting time (CT) remained normal in all the envenomed dogs. Coagulation properties have not been well recognized in *N. naja* venom [33]. AVS which contains polyclonal antibodies—Fab or F(ab)2 fragments—is a refined and concentrated preparation of equine serum globulins obtained by fractionating blood from healthy horses that have been immunized against snake venoms [5,34]. Indian polyspecific antivenom consists of F(ab)2 antibody, with a half-life between 80–100 h [22]. Indian polyvalent AVS contains horse-derived F(ab)2 antibody fragments derived from *Bungarus caeruleus*, *D. russelii*, *Echis carinatus,* and *N. naja* from India [27,35,36]. Polyvalent AVS consists of a mixture of antibodies that share common antigenic regions against different constituent toxins. In fact, they have the ability of cross neutralization for a broad range of snake venom toxins [5,37,38]. The currently available Indian polyvalent AVS is efficacious in preventing envenoming in dogs in Sri Lanka despite the AVS-induced adverse effects. Inter- and intraspecies variability of venom composition of the relevant snakes are factors affecting the effectiveness of the AVS. Consequently, in securing the data quality of clinical studies, the case definition is of the utmost importance [39]. This study has shown that the number of AVS vials that should be administered can be determined according to the visual scoring scale. These can be obtained with the help of three criteria from the site of the bite. Local and systemic clinical manifestations are statistically significant (probability value < 0.05 for all the groups for probit analysis). Interestingly, the scoring system established is a simple, time-saving, and cost-effective method which clinicians are able to apply as a bedside evaluation method to determine the number of AVS vials that must be used to initiate the treatments for cobra-bite-envenomed dogs.

The extent of envenoming is directly associated with the site of envenoming, according to a study conducted in France [40]. Snakebite envenoming, which is common in tropical rural developing geographical settings, in both man and animal is categorized as a medical emergency. Moreover, the final outcome of this incidence may result in permanent disability or death [22]. Therefore, these patients must be evaluated, treated, and managed quickly in order to safeguard their lives. However, there are no specific technical facilities or expertise available to quantify the venom concentration in snakebite victims in the majority of rural clinical settings. Further, quantification techniques are time consuming and lead clinicians to delay initiating AVS treatments, which may result in a serious deterioration in the patient’s condition. Dogs are inquisitive by nature and prone to multiple bites [8]. Therefore, the scoring formula developed in this study will definitely be an aid to measuring the level of envenoming in dogs. Moreover, the scoring system is simple and easy to apply to evaluate the level of envenoming; it is thus user friendly for clinicians.

Anaphylactoid and anaphylaxis reactions to AVS are reported in around 80% of snakebite victims, with Fc fragments resulting in papain digestion and IgG aggregates due to mast cell activation [13,27]. In order to manage antivenom-related anaphylaxis, adrenaline, hydrocortisone, and chlorphenamine are recommended, though the exact mechanism and the advantages of these precautionary measures have not yet been explored [35,41,42]. Unlike in humans, as bite wounds are likely to be contaminated with soil and dirt, secondary bacterial infection of wounds is a realistic possibility and therefore antibacterial agents such as cloxacillin and metronidazole are routinely initiated [13]. In order to maintain the hydration status and metabolic activities of the body, water electrolyte and adequate energy levels must be maintained with parenteral normal saline, lactated Ringer’s solution, and dextrose as indicated.

Adverse reactions to AVS in cobra-bite-envenomed dogs occurred proportionately in this study. Adverse reactions such as acute lung injury must be treated in an appropriate setting with sufficient facilities because cobra venom may aggravate this condition due to its toxins causing neuromuscular paralysis [5]. In critical situations, acute lung injuries are managed under light anesthesia in order to facilitate respiration by means of intubation [13]. An intensive care unit (ICU) with a trained technical staff, safe anesthetics, and other technical facilities are key to safeguard envenomed dogs. AKI in snake envenoming is a multifactorial consequence due to direct nephrotoxicity of venom coagulopathy, hypotension, disseminated intravascular coagulation, intravascular hemolysis, thrombotic microangiopathy, “capillary leak syndrome”, rhabdomyolysis, complement activation, and secondary sepsis [43,44]. In this study, cobra-bite-envenoming-induced AKI was recorded in a small proportion of the dogs. However, assessment of kidney function is vital to determine impending renal injuries. The severity of signs and symptoms of envenoming greatly depends on the location of the fang marks. All the patients (3.4%) that died due to cobra bite envenoming had fang marks on their oral cavity, including the lips, tongue, and gums. Therefore, if a dog is bitten at its oral cavity, there is a great risk of fatality. This could be because this anatomical site is highly vascularized and close to the central nervous system. Subsequently, these features may facilitate the spread of toxins more rapidly, leading to prompt envenoming. Hence, dogs presenting with fang marks on their oral cavity must be considered the most vulnerable and, therefore, must be treated aggressively.

## 4. Conclusions

In order to assess the severity of envenoming, a proposed scoring system is suggested, comprising the parameters of visible location of fang marks and local and systemic clinical profiles which have proved to be effective in deciding the number of Indian polyvalent AVS vials to be administered to dogs. Statistical analysis has proved that visible grading scores and the number of AVS vials required to treat each severity group are compatible. The formula created can be applied to evaluate the level of envenoming in a simple and rapid way. Further, cobra bite envenoming causes necrosis and neurotoxic effects in both humans and animals, where venom concentration cannot be measured rapidly. This gradation may be applied to assess the level of envenoming in both companion animals and farm animals. Therefore, the findings of this study may be used as guidance in rural underdeveloped geographical settings not only in Sri Lanka but also throughout the world, as many primary care settings lack modern technical facilities. As AVS-induced adverse reactions are abundant, the clinician must be mindful to manage such critical situations. Dogs that are highly excited must be taken into a peaceful environment in order to calm them, slowing the spread through the circulation of the venom. The development of acute renal failure (ARF) can be reversed if the patients are monitored intensively for complications. Consequently, the administration of AVS was mainly based on qualitative parameters in this study; thus, it is of utmost importance to focus on further studies to develop techniques for identification and quantification of venom in envenomed dogs.

## 5. Materials and Methods

### 5.1. Study Setting and Data Collection

A prospective study was conducted on cases admitted to VTH, University of Peradeniya, with the complaint of dogs with cobra (*N. naja*) bite for 72 months between January 2012 and December 2017. Distinctive features of the offending snakes were collected from dog owners. Envenomed dogs were examined and treated in the intensive care unit (ICU), as it provides a calm environment. Physical and psychological methods of restraint were adopted for general clinical examination, patient preparation for blood and urine sampling, and administration of therapeutic agents. On admission of all patients, demographic information and significant details (breed, sex, age, and body weight) were recorded. Identification of the location of fang marks was carried out by the researcher with the help of the attending clinician.

### 5.2. Recognition of Offending Snake

Offending snakes were recognized by one of three methods. Dead or live snake specimens brought by dog owners were compared to published characteristics of native snakes [8]. Photographs of the culprit snakes were used (often taken by mobile phone). When neither specimens nor photographs were available, owners were asked to compare the other photographs and preserved specimens to the culprit snakes for tentative snake identification.

### 5.3. General Clinical Examination

All the body systems of the victim were examined, and qualitative and quantitative measurements of the clinical signs and symptoms were recorded. In addition to this routine examination, a detailed history of the snakebite incident was obtained from the owner. Information regarding the anatomical location/s of the sites of the bite and the number of fang marks was gathered and graded as shown in Table 5.

**Table 5:** Grading of snakebites according to anatomical location/s in dogs reported to the Veterinary Teaching Hospital (VTH).

Grade	Anatomical Location	Score
Grade 1	Head and neck	3
Grade 2	Limbs	2
Grade 3	Thorax and abdomen	1

Clinical manifestations of local envenoming of pain at the site of the bite, extent of swelling, and hemorrhagic blister or necrotic areas were graded. Scores were given accordingly from 1–3 as indicated in Table 6.

The parameters of body temperature, heart rate, pulse rate and respiratory rate of the victim were recorded every 15 min for 2 h and thereafter hourly for 6 h. However, patients that needed continuous care were monitored intensively until they had stabilized. Neurological manifestations; abnormalities in respiratory, cardiovascular, and urogenital systems; and gastrointestinal signs and symptoms were observed and graded according to their manifestations (Table 7).

According to the studies conducted by Audebert et al., significant correlation was found between the clinical manifestations of envenoming and the level of venom in blood or urine. Another study conducted to assess the epidemiological pattern of hump-nosed viper (*Hypnale hypnale*) envenoming among children and adults was adopted to establish grading criteria [44]. According to the AVS manufacturer’s guidelines, a minimum single AVS vial must be administered to the patient, as it has the ability to neutralize 4 mg of venom [22]. Another study conducted in VTH demonstrated that many dogs are encountered with multiple bites on the head and neck region. These findings were used to establish grading scores [8]. In this study, the grading score for each patient was given according to the equation provided below:

Grading score (*i*) = Anatomical location of the fang marks (*j*) + Local clinical manifestations (*k*) + Systemic clinical manifestations (*l*) (1)

where *i* can vary in the range of 1–17 according to the severity inflicted (Table 8), *j* in the range of 1–6 (Table 5), *k* in the range of 1–6 (Table 6), and *l* in the range of 0–5 (Table 7).

### 5.4. Laboratory Procedures

Blood and urine samples were collected on admission, 24 h postadmission, and daily until the patient was discharged. Collected blood samples were tested for full blood count (FBC), BUN, creatinine, alanine aminotransferase (ALT), aspartate aminotransferase (AST), total protein, prothrombin time (PT), activated partial thromboplastin time (aPTT), and whole blood clotting time (CT). Repeated testing of PT, aPTT, and CT were performed every 6 h after admission until they returned to normal values. A urine full report (UFR) was obtained on admission and 24 h after treatment. In addition, urine output was calculated for all patients. Daily UFR was performed to assess functional activities of the urinary system. When an aberrant test result was seen, the treatment was adjusted to correct the abnormality.

### 5.5. Initiation of Treatments—Auxiliary Treatments

Cobra bite envenoming is considered a medical emergency. Therefore, prompt action was taken to establish two simultaneous intravenous access sites for all patients once the victims were taken into ICU. As auxiliary treatment, adrenaline (Adrivit 0.1% *w*/*v*, 1 mL, Healthcare Pvt. Ltd., New Delhi, India: 0.01–0.02 mg/kg, s.c., stat.) and hydrocortisone (hydrocortisone sodium succinate for injection BP 100 mg, AMN Life Science Pvt Ltd., Mumbai, India: 10 mg/kg, IV, q.i.d.) simultaneously with AVS, chlorpheniramine maleate (Allervit 1% *w*/*v*, 1 mL, Healthcare Pvt. Ltd., New Delhi, India) 0.4 mg/kg, IV, q.i.d.), cloxacillin sodium (for injection BP 250 mg, Vysali Pharmaceuticals Limited, Kerala, India: 20 mg/kg, IV, b.i.d.), metronidazole (Metronidazole Intravenous Infusion BP 500 mg/100 mL, Claris Life Sciences Limited, Ahmedabad, India: 20 mg/Kg, IV, b.i.d.), and fluids (normal saline, lactated Ringer’s solution, 10% dextrose in appropriate volumes) were administered intravenously for the initial 24 h of hospitalization. These treatments were continued until the patients recovered. Where necessary, changes to the treatment schedule were undertaken depending on the requirements of the patient.

### 5.6. Determining the Indication and Treatment with Indian Polyvalent AVS

In order to decide the number of vials of AVS to be administered on the initiation of AVS treatment, the following criteria were used. The scoring system (Table 8) was established based on the anatomical location/s of fang marks, local and systemic envenoming manifestations [1,12], and the manufacturer’s guidelines. AVS (Anti Snake Venom Serum I.P.—10, (I.V. mL/ Lyophilized^TM^, VINS Bioproduct Ltd., Hyderabad, India)) was used to treat envenomed dogs. According to the scores that a particular patient obtained, the appropriate number of AVS vials was administered. Each vial, with the ability to neutralize 6 mg of venom antigen, was dissolved in 10 mL of normal saline. Then, the dissolved AVS was added to normal saline at the ratio of 1:1 and administered at the rate of 2 mL/min. Continuation of further AVS treatment was decided upon the results of the laboratory tests and clinical manifestations of the patient.

### 5.7. Management of Complications/Adverse Reactions Induced by AVS

Any clinical sign observed during and after administration of AVS was considered as an adverse reaction to the AVS. Therefore, patient monitoring for the development of adverse reactions was initiated immediately on AVS administration. Hypersalivation, fever, restlessness, itching, dyspnea, tachypnea, wheezing, stridor, crackles, hypotension, bradycardia, tachycardia, and laryngeal edema are the clinical manifestations observed as adverse reactions for Indian polyvalent AVS in dogs [13]. Dogs observed with signs of hypersensitivity were treated with adrenaline, chlorpheniramine maleate (0.4 mg/kg, IV, q.i.d.), and hydrocortisone (10 mg/kg, IV, q.i.d.) to minimize the activation of immune mediators (histamine, prostaglandin, leukotriene, cytokines, etc.). Atropine sulphate (Atrover 0.6 mg/mL ampule, Veve Human Care Laboratories, India: 0.02 mg/kg, IV, stat.) was administered to avert hypersalivation. Any type of cardiovascular manifestation (bradyarrhythmias and hypotension) was corrected by administration of inotropes intravenously with dopamine (Ciron Drugs & Pharmaceuticals Pvt. Ltd. Nagarrabhat Nagar, Ground Floor, Jogeshwari (West) Mumbai, India: 5 mcg/kg/min) and adrenaline (0.01–0.02 mg/kg, stat.).

If any patient displayed respiratory difficulty, it was examined for possible laryngeal edema and airway obstruction due to hypersalivation, aspiration, secretions, and so forth. Respiration was assisted in such patients using endotracheal intubation. Any conscious patient that was observed with deteriorating signs of dyspnea was sedated with diazepam (Calmvita 5 mg/mL, Healthcare Pvt Ltd., New Delhi, India: 0.5 mg/kg, IV, stat.) and, later, mild anesthesia was induced with the lowest dose rate of propofol hydrochloride (Anesia^TM^ 1% Injection for Intravenous Infusion 50 mL/vial, Claris Life Sciences Limited, Ahmedabad, India: 4 mg/kg, IV, stat.). Clinical manifestation of respiration was assessed with the color of the mucosal membranes, auscultation of the respiratory system, and by pulse oximeter. Cardiovascular signs were monitored and patients with hypotension were treated accordingly. Initially, such patients were treated with adrenaline (0.01–0.02 mg/kg, stat.) and dopamine (5 mcg/kg/min).

### 5.8. Assessment of Kidney Function and Management of Acute Kidney Injuries (AKIs)

Insufficient renal function was diagnosed through observation of clinical manifestations and laboratory investigation of UFR together with urine output (UO). Patients that developed anuria or oliguria were treated with furosemide (Furosemide 20 mg/2mL Solution For Injection, Steril-Gene Life Sciences (P) Ltd., Chennai, India: 2 mg/kg IV, 4 mg/kg, or 6 mg/kg IV), administered at standard intervals (a minimum of 30 min elapsed between each furosemide dose) until urine production was observed. Once a patient responded to the treatment, furosemide (3 mg/kg, IV b.i.d.) was continued until the urine production returned within the normal range (UO > 66%).

### 5.9. Tackling the Cobra-Bite-Induced Local Tissue Damage

**Figure 4:**
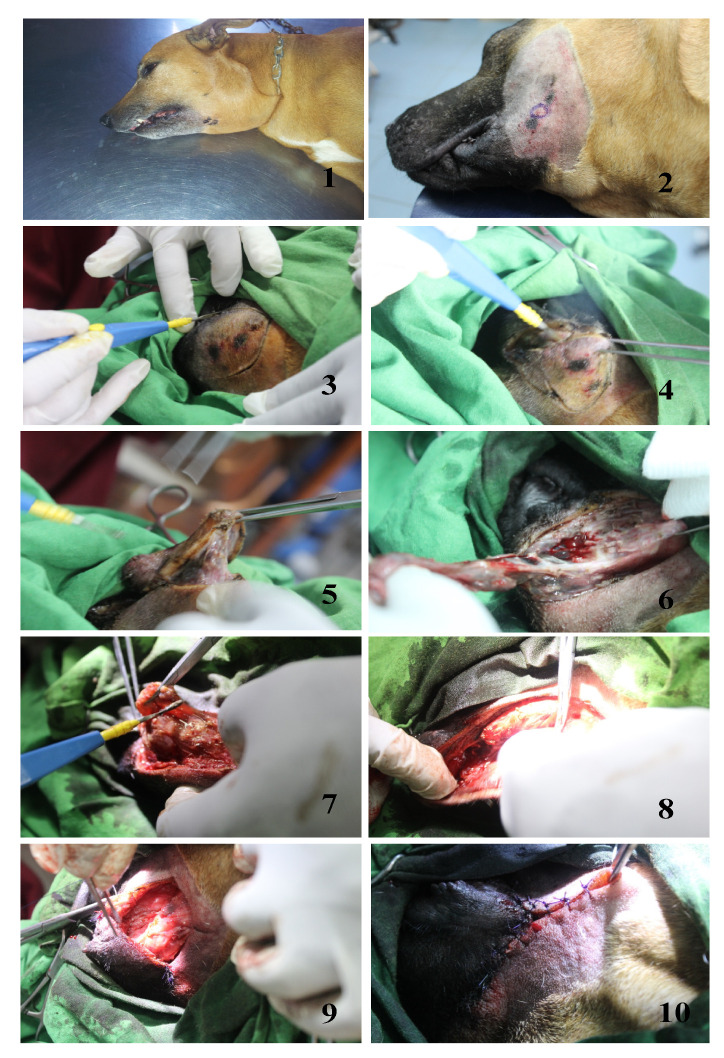
Surgical excision of necrotic tissues to minimize extent of trauma.

Wounds with necrotic tissue, when observed, were treated with the aim to reduce the amount of necrotic tissue and further aggravation of necrosis. In some cases, extensive swelling at the site of the bite worsened over time. In some dogs, pus-filled necrotic areas were covered with necrotic skin that eventually sloughed off. Such individuals were sedated with diazepam (0.5 mg/kg, IV, stat.) and then anesthesia was induced with propofol hydrochloride (6.6 mg/kg, IV), maintaining propofol hydrochloride administration throughout the procedure. Necrotic skin and underlying tissues (fascia and muscles) were excised, and wounds were cleaned with double-diluted hydrogen peroxide and normal saline. An incision was made in the most dependent part of the edematous area and a Penrose drain was applied to drain out the purulent material. Wound cleaning with 0.9% normal saline and the application of povidone iodine solution was repeated daily until sutures could be applied. The patients were anesthetized using the same procedure mentioned above. The wound was cleaned with normal saline and the edges of the wound were scarified until bleeding. Subcutaneous tissue (with 2/0 chromic catgut) and skin (with suture nylon) were sutured with a simple interrupted suture pattern. The same antibiotic combination (cloxacillin 20 mg/kg, IV, b.i.d. and metronidazole 20 mg/Kg, IV, b.i.d.) was continued until complete recovery of the patient (Figure 4).

### 5.10. Statistical Analysis

Data documentation and descriptive statistics were employed using Microsoft Excel (Microsoft, Redmond, WA, USA) and MINITAB 16 software (Minitab Inc.: State College, PA, USA). In order to assess the number of AVS vials given to the envenomed dogs upon initiation of treatment based on visual clinical signs and symptoms, a probit analysis for the survival percentage in the dataset from the 1st to the 12th severity group was performed. The statistical method was a binary logistic regression in SAS (SAS Institute Inc., Cary, NC, USA).

## 6. Declaration

### 6.1. Ethics Approval

Ethical clearance was obtained from the Ethical Review Committee of the Faculty of Veterinary Medicine and Animal Science, University of Peradeniya, Sri Lanka (Ref No.VER-14-012, 17 December 2014), which is on par with the international standards of ethics on animal experimentation. All experimental procedures and animal care were approved by the Faculty Ethics Committee, Faculty of Veterinary Medicine and Animal Science, University of Peradeniya, Sri Lanka.

### 6.2. Consent for Publication

We certify this manuscript has not been published elsewhere and is not submitted to another journal. All authors have approved the manuscript and agreed with submission to The Journal of Toxins.

### 6.3. Availability of Data and Material

The datasets and materials for this study have been retained.

## Figures and Tables

**Table 1:** Patient number according to severity of bite, scoring obtained, vials of antivenom (AVS) used, and deaths recorded.

Grading Score	Number of AVS Vials Given	Number of Patients	Percentage of Patients (%)	Deaths Percentage
1–2	1	0	0	0
3–4	2	10	8.6	0
5–6	3	20	17.2	0
7–8	4	12	10.2	0
9	5	36	31.0	0.86
10	6	16	13.7	1.7
11	7	6	5.3	0
12	8	0	0	0
13	9	4	3.3	0.86
14	10	6	5.3	0.86
15–17	12	6	5.3	0

**Table 2:** Clinical manifestations observed in *N. naja* envenomed dogs.

Local Effects	Number of Patients	Percentage
Pain	112	97
Mild swelling	51	44
Swelling	36	31
Necrosis	22	19
**Neurological Effects**		
Hypersalivation	61	53
Mydriasis	61	53
Wheezing, crackles	55	47
Tachypnea	74	64
Bradypnea	16	14
Wheezing	55	47
Bradycardia	20	17
Cardiac arrhythmias	67	58
Hypotension	26	22
**Other Effects**		
Vomiting	7	6
Diarrhea	13	11
Recumbency	38	33
Hyperesthesia	49	42

**Table 3:** Abnormalities detected in laboratory investigations at admission and after 24 h in cobra-bite-envenomed dogs.

Parameter	Patients % on Admission	Patients % on 24 h of Admission
Leukocytosis	78	94
Thrombocytopenia	33	44
	13	6
Proteinuria	11	6
PT	17	0
aPTT	25	0

PT: prothrombin time; aPTT: activated partial thromboplastin time.

**Table 4:** Abnormalities observed in full blood count (FBC), PT, and aPTT in cobra-envenomed dogs reported.

Character	% of Dogs	Minimum	Maximum	Mean	Median	IQR
Leukocytosis (×10^3^/mL)	78	21.81	80.50	40.54	34.61	19.76
Neutrophilia (%)	78	75.90	92.20	82.04	82.05	7.30
Anemia (%)	8	15.7	25.9	22.33	23.25	5.18
Thrombocytopenia (×10^3^/mL)	33	97	190	138.9	130.05	64.8
Elevated PT (seconds)	17	11.5	30.00	17.89	16.30	10.35
Elevated aPTT (seconds)	25	22.4	68	32.22	28.70	10.78

IQR: interquartile range.

**Table 6:** Grading of local effects of cobra bite envenoming in dogs reported to VTH.

Grade	Features	Score
Grade a	Mild swelling	1
Grade b	Extensive swelling	2
Grade c	G2 + necrosis	3

**Table 7:** Grading of systemic manifestation of cobra bite envenoming in snake-envenomed dogs reported to VTH.

Grade	Feature	Score
Grade A	Neurological manifestations	1
Grade B	Respiratory manifestations	1
Grade C	Cardiovascular manifestations	1
Grade D	Urogenital manifestations	1
Grade E	Gastrointestinal manifestations	1

**Table 8:** Suggested number of AVS vials to be given according to scores obtained.

Score	Number of AVS Vials for Initial Dose
1–2	1
3–4	2
5–6	3
7–8	4
9	5
10	6
11	7
12	8
13	9
14	10
15–17	12
